# Response to: Comment on “The Use of Pulsed Electromagnetic Fields to Promote Bone Responses to Biomaterials In Vitro and In Vivo”

**DOI:** 10.1155/2020/9801420

**Published:** 2020-09-29

**Authors:** G. Pedrazzi, C. Galli, M. Mattioli-Belmonte, S. Guizzardi

**Affiliations:** ^1^Department of Medicine and Surgery, University of Parma, Parma, Italy; ^2^DISCLIMO, Department of Clinical and Molecular Sciences, Polytechnic University of Marche, Ancona, Italy

The following is a reply to the letter to the editor sent by Ramirez-Vasquez et al. (2019) concerning the reported values of the magnetic fields published in our article “The use of pulsed electromagnetic fields to promote bone responses to biomaterials *in vitro* and *in vivo*” (2018). Since our original paper may be of interest to a larger audience than only physicists or engineers, we believe that some introductory remarks are necessary to properly understand the issue.

**The Electromagnetic Spectrum**

It is well known that the world of electric and magnetic fields—how they interact, propagate, and how they are affected by objects—can be described by the so-called Maxwell's equations. These are a set of four coupled and complex equations that form the foundation of classical electromagnetism. Maxwell's equations provide a mathematical framework for all modern technologies, such as power generation, electric motors, wireless communication, lenses, and radar. The existence of electromagnetic (EM) waves (or “radiation”) was first inferred from the solutions to Maxwell's equations, years before they were experimentally proven. All electromagnetic waves are solutions to Maxwell's equations. Waves with different frequencies, however, exhibit very different behaviors when they interact with matter.  Figure 1 summarizes the so-called EM spectrum. The highest frequency part of the spectrum is made up of types of ionizing radiation, e.g., *γ*-rays (gamma radiation), X-rays, and ultraviolet (UV) radiation. These radiation types are capable of extracting electrons from atoms and turning them into electrically charged ions, which may cause radiation damage. Waves with lower frequencies, e.g., visible light or radio waves, cannot ionize atoms and are thus known as nonionizing radiation.

If we move to the lower frequencies of the spectrum, the common solutions of Maxwell's equations are no longer ordinary EM waves. Therefore, the term used to describe the frequencies around 50 or 60 Hz is low-frequency electric and magnetic “*fields*,” in short, electromagnetic fields (EMFs). EMFs do not integrally belong to the electromagnetic spectrum. We decided to include “power line radiation” in parentheses in Figure 1 for this reason.

When we refer to the intensity, dose, and biological effects of ionizing radiation, it is relatively easy to characterize the radiation intensity at various positions in space and its direction. When ionizing radiation and light—which both have a high frequency—are considered, their wavelength is always extremely small compared to cells or living organisms, but the same cannot be said for low-frequency fields. These may vastly differ, according to the source that generated them, and their biological effect may depend on a more complex set of parameters than with ionizing radiation, thus posing a serious challenge to investigators trying to characterize them.

**Near Fields vs. Radiating Fields/Waves**

EM fields are usually classified as static, extremely low frequency, very low frequency, radiofrequency, or microwave, according to the frequency with which they vary over time. Higher frequencies are regarded as electromagnetic waves (“radiation”). Although there is some disagreement on how to classify EMFs, one possible distinction is presented in Table 1.

A similar characterization is used for higher frequency electromagnetic waves. It is however common practice to distinguish between “fields” and “waves.”

Electromagnetic waves (Figure 2) are particular solutions of Maxwell's equations with specific properties:

- The electric and magnetic fields are perpendicular to each other and to the direction of propagation.
- There is a unique relationship between the magnitudes of the electric and magnetic fields so that it is possible to compute one based on the other.
- An EM wave carries energy away from the source, and usually, the energy does not return to that source.
- There is a definite relationship between frequency, *f*, and wavelength, *λ*: *f* ·*λ* = *c*, where *c* is the speed of light.
- At least for waves with *λ* similar to visible light, the energy in the EM wave is exchanged with its surroundings by photons. Each photon has an energy *E* = *h*·*f,* where *h* is the Planck constant and *f* is the wave frequency.

However, it is very important to note that *none* of the characteristics that were just mentioned above hold true for low-frequency EMFs. This is a very important aspect. Their electric and magnetic fields are not closely related. When assessing EMFs around power lines, electric appliances, etc., it is possible to detect strong electric fields and very weak magnetic fields or even just the opposite. The electric and magnetic fields are not even necessarily perpendicular to each other, they do not carry away energy, and the fields do not exhibit wave behavior; it therefore does not make sense to talk about wavelengths in these situations.

As an approximate rule, we can say that wave characteristics dominate the fields when viewed farther than about one calculated wavelength away from the source. The calculated wavelength can be simply found as *calculated wavelength* = *speed of light/frequency*.

This means that, when visible light is considered, wave behavior is encountered at a distance larger than about 500 nm from the source. This distance however goes up to 330 mm for a 900 MHz mobile telephone, and a staggering 6000 km is necessary for a 50 Hz appliance or power line. As an analogy, the electric and magnetic fields generated by power lines here on Earth would be found to follow the characteristics of electromagnetic waves on the Moon, assuming that we had sensitive instruments capable to pick up this signal from that distance. However, for every practical situation on Earth, the wave part of these fields is negligible. The term “the near field” is used to denote this situation, as opposed to “the far field,” where wave behavior dominates [2].

In the near field, the electric and magnetic fields might be almost independent of each other.

As energy in the near fields cannot be said to be transported away from the source, researchers in this area do not tend to use the term “radiation,” but rather resort to, as it was done in this letter, the term *fields*: electric fields and magnetic fields or “*electromagnetic fields*” to include both.

Concerning the comments by Ramirez-Vasquez et al. [1], they raise some doubts about the reported values of magnetic fields because of the calculated value of the intensity of the electromagnetic wave according to the formula *I*=*cB*_max_^2^/2*μ*_0_ (W/m^2^).

We have at least three objections to their concerns:

1. As stated above, the range of frequencies we are dealing with in the studies we reviewed are in the extreme low frequency range or very low frequency range, and we are thus facing a “near field situation.” As reported also in the guideline of the International Commission on Non-Ionizing Radiation Protection (ICNIRP—Health Physics 74 (4):494–522; 1998, page 45), “The situation in the near-field region is rather more complicated because the maxima and minima of *E* and *H* fields do not occur at the same points along the direction of propagation as they do in the far field. In the near field, the electromagnetic field structure may be highly inhomogeneous, and there may be substantial variations from the plane-wave impedance of 377 ohms; that is, there may be almost pure *E* fields in some regions and almost pure *H* fields in others. Exposures in the near field are more difficult to specify, because both *E* and *H* fields must be measured and because the field patterns are more complicated; in this situation, power density is no longer an appropriate quantity to use in expressing exposure restrictions (as in the far field).” [3, 4].
2. ICNIRP restriction values begin to report power density (W/m^2^) at frequencies of the order or higher than those of radio waves.
3. Therefore, the intensity calculation of Ramirez-Vasquez et al. [1] is not appropriate for the present case.
4. The magnetic fields reported in our review all come from studies where the authors directly measured the magnetic fields using appropriate instruments. We have no reasons to doubt the correctness of the measurements, which are also very consistent across the literature.
5. The magnetic field values reported in the paper are of the same order of magnitude as the values measured around some domestic appliances, e.g., hairdryers or electric razors (Table 2). To the best of our knowledge, no one has ever raised serious concerns about these devices.

On the other hand, all the studies on model biological systems reported in our review [5] aim to demonstrate a possible role of applied electromagnetic fields in bone responses or healing processes. Necessary caution should be applied when using such tools, and further research should be conducted to ascertain that their use is safe, although exposure may be limited to a single affected body part and the duration of the investigated treatments is generally limited.

## Figures and Tables

**Figure 1:**
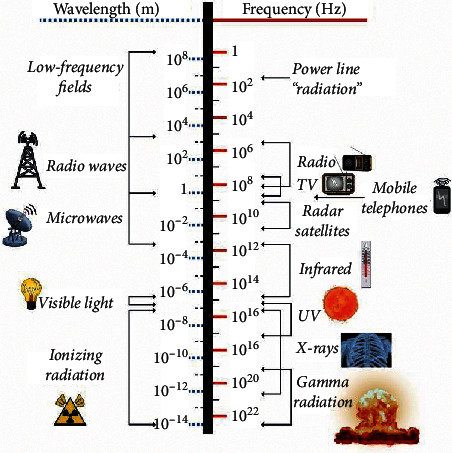
Electromagnetic spectrum.

**Figure 2:**
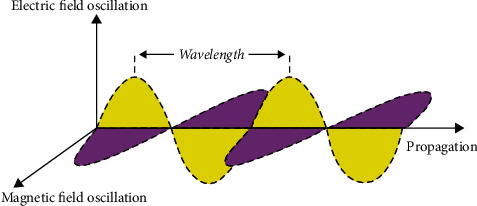
This is a common representation of electromagnetic waves. They are particular solutions of Maxwell's equations, but in practice they occur only for radio waves and higher frequencies. In proximity to power lines and appliances, where only static or low-frequency currents or voltages occur, the electric and magnetic fields do not follow the characteristics given in this figure.

**Table 1:** Classification bands for low-frequency electromagnetic fields.

Classification	Abbreviation	Frequency band
Static fields	—	0–3 Hz
Extreme low frequency	ELF	3–3000 Hz
Very low frequency	VLF	3–300 kHz
Radiofrequency	RF	0.3–300 MHz
Microwaves	—	0.3–300 GHz

**Table 2:** Typical values for the magnetic fields generated by some commonly used electrical appliances.

Electrical appliances	Distance: 3 cm (*μ*T)	Distance: 30 cm (*μ*T)	Distance: 1 m (*μ*T)
Hairdryers	6–2000	0.01–7	0.01–0.03
Electric razors	15–1500	0.08–9	0.01–0.03
Vacuum cleaners	200–800	2–20	0.13–2
Fluorescent lamps	40–400	0.5–2	0.02–0.25
Microwave ovens	73–200	4–8	0.25–0.6
Radios	16–56	1	<0.01
Electric ovens	1–50	0.15–0.5	0.01–0.04
Washing machines	0.8–50	0.15–3	0.01–0.15
Irons	8–30	0.12–0.3	0.01–0.03
Dishwasher	3.5–20	0.6–3	0.07–0.3
Computers	0.5–30	<0.01	—
Refrigerators	0.5–1.7	0.01–0.25	<0.01
Color TV	2.5–50	0.04–2	0.01–0.15

The intensity of the field does not depend on the size, complexity, power, or noise of the device. Moreover, magnetic field strength can vary greatly even between apparently similar devices. For example, some hairdryers may be surrounded by a very intense field, while others generate an almost negligible magnetic field, depending on the design of the apparatus. The measurements were carried out in Germany (from: Federal Office for Radiation Security, Germany 1999), and all the appliances operate on electricity with a frequency of 50 Hz. It should be noted that the actual levels of exposure vary considerably depending on the model of the appliance and the distance from it.
